# Serotype distribution, antimicrobial susceptibility and molecular epidemiology of invasive *Streptococcus pneumoniae* in the nine-year period in Serbia

**DOI:** 10.3389/fmicb.2023.1244366

**Published:** 2023-08-21

**Authors:** Natasa Opavski, Milos Jovicevic, Jovana Kabic, Dusan Kekic, Zorica Vasiljevic, Tanja Tosic, Deana Medic, Suzana Laban, Lazar Ranin, Ina Gajic

**Affiliations:** ^1^Faculty of Medicine, Institute of Microbiology and Immunology, University of Belgrade, Belgrade, Serbia; ^2^Department of Clinical Microbiology, Mother and Child Health Care Institute of Serbia "Dr. Vukan Cupic", Belgrade, Serbia; ^3^Department of Microbiology, University Clinical Center of Serbia, Belgrade, Serbia; ^4^Faculty of Medicine, University of Novi Sad, Novi Sad, Serbia; ^5^Center for Microbiology, Institute of Public Health of Vojvodina, Novi Sad, Serbia; ^6^Department of Microbiology, University Children's Hospital, Belgrade, Serbia

**Keywords:** *Streptococcus pneumoniae*, serotype, antimicrobial susceptibility, molecular epidemiology, Serbia

## Abstract

*Streptococcus pneumoniae* is one of the leading bacterial pathogens that can cause severe invasive diseases. The aim of the study was to characterize invasive isolates of *S. pneumoniae* obtained during the nine-year period in Serbia before the introduction of the pneumococcal conjugate vaccines (PCVs) into routine vaccination programs by determining: serotype distribution, the prevalence and genetic basis of antimicrobial resistance, and genetic relatedness of the circulating pneumococcal clones. A total of 490 invasive *S. pneumoniae* isolates were included in this study. The serotype, antimicrobial susceptibility, and ST of the strains were determined by the Quellung reaction, disk- and gradient-diffusion methods, and multilocus sequence typing (MLST), respectively. The most common serotypes in this study were 3, 19F, 14, 6B, 6A, 19A, and 23F. The serotype coverages of PCV10 and PCV13 in children less than 2 years were 71.3 and 86.1%, respectively, while PPV23 coverage in adults was in the range of 85-96%, depending on the age group. Penicillin and ceftriaxone-non-susceptible isolates account for 47.6 and 16.5% of all isolates, respectively. Macrolide non-susceptibility was detected in 40.4% of isolates, while the rate of multidrug- and extensive-drug resistance was 20.0 and 16.9%, respectively. The MLST analysis of 158 pneumococci identified 60 different STs belonging to the 16 Clonal Complexes (CCs) (consisting of 42 STs) and 18 singletons. The most common CC/ST were ST1377, CC320, CC15, CC273, CC156, CC473, CC81, and CC180. Results obtained in this study indicate that the pre-vaccine pneumococcal population in Serbia is characterized by high penicillin and macrolides non-susceptibility, worrisome rates of MDR and XDR, as well as a high degree of genetic diversity. These findings provide a basis for further investigation of the changes in serotypes and genotypes that can be expected after the routine introduction of PCVs.

## 1. Introduction

*Streptococcus pneumoniae* remains one of the leading community-acquired bacterial pathogens worldwide. The spectrum of pneumococcal diseases ranges from non-invasive, such as acute otitis media (AOM), sinusitis, and non-bacteremic pneumonia, to invasive as bacteremic pneumonia, sepsis, and meningitis. Young children and the elderly are the most affected age groups, with more than 300,000 deaths annually attributable to *S. pneumoniae* in children younger than five years (Wahl et al., 2018) and more than 690,000 deaths in adults over 70 years in 2015 worldwide (Troeger et al., 2017). However, invasive pneumococcal disease (IPD) dramatically declined in children and adults following the introduction of pneumococcal conjugate vaccines (PCVs) (CDC, 2019). Due to the phenomenon of serotype replacement and the emergence of non-vaccine serotypes, higher-valent PCVs (PCV10, PCV13, PCV15, and PCV20) replaced first-generation vaccines (PCV7). By the end of 2020, 148 out of 194 WHO member states have introduced PCV into their National Immunization Programs (NIPs) (CDC, 2020). Although the PCV7 was licensed in Serbia in 2009, and PCV10 and PCV13 in 2013, they have been available on a voluntary basis, and the coverage rate of PCVs was very low up to 2018. Thereafter, PCV10 began to be used routinely for immunization of children under 2 years of age in 2018,^1^ and in 2022 it was replaced by PCV13. PCV10 vaccine coverage in 2018 did not exceed 50%.^2^ In the period 2018-2021, vaccine efficacy and effectiveness and the potential serotypes shift could not be well monitored due to the COVID-19 pandemic and subsequently reduced number of IPD cases and low quality of IPD monitoring. The pneumococcal polysaccharide vaccine (PPV23) has been available in Serbia for over 20 years for risk groups.

Reliable epidemiological surveillance of infectious diseases is critical for making rational decisions on public health issues such as vaccination strategies. The choice of PCV to be used in a country should be primarily based on the local and regional prevalence of vaccine serotypes, antimicrobial resistance patterns, vaccine availability, vaccine cost, etc. (World Health Organization, 2021). In Serbia, IPD is notifiable according to the current Law on Protection of the Population from Infectious Diseases.^3^ Despite the legislative obligation, in practice, it is not fully implemented, and data on the incidence of IPD in our country is scarce. So far, no population-based study on IPD has been performed in Serbia. However, National Reference Laboratory (NRL) for Streptococci performs voluntary-based laboratory surveillance on IPD and serotype distribution (Gajic et al., 2013; Delic et al., 2021).

Antibiotic treatment of pneumococcal infections typically includes beta-lactams and macrolides, in addition to fluoroquinolones in adults. Due to the high antibiotic consumption in Serbia (Tomas et al., 2021) as well as very common non-invasive pneumococcal diseases that are treated with antibiotics, the rate of macrolide resistance among the invasive pneumococci in Serbia in 2020 was 31.8%, while up to 48.1% of isolates expressed decreased susceptibility to penicillin (WHO Regional Office for Europe/European Centre for Disease Prevention and Control, 2022). It is known that clonal expansion is considered to be the main mechanism for ongoing antibiotic resistance (Keenan et al., 2015). Also, the pneumococcal population structure is highly diverse, varies between geographic locations, and evolves and adapts to the selective pressure of PCVs (Lo et al., 2019). Therefore, information on the genetic background in circulating *S. pneumoniae* isolates will allow us to analyze further changes as a result of the implementation of PCV in Serbia.

Continuous surveillance of IPD is important both prior to and after vaccine introduction. Serotype distribution in the pre-vaccine period provides a baseline for evaluating the impact of PCV on *S. pneumoniae* epidemiology. Therefore, this study aimed to characterize invasive isolates of *S. pneumoniae* obtained up to the introduction of the PCVs in the Serbian NIP by determining: serotype distribution, the prevalence and genetic basis of antimicrobial resistance, and the genetic relatedness of the circulating pneumococcal clones.

## 2. Materials and methods

### 2.1. Bacterial collection, identification, and conservation of pneumococcal isolates

This study included all invasive *S. pneumoniae* isolates obtained from the regional microbiological laboratories of hospitals and clinical centers throughout Serbia, a Southeastern European country with roughly 6,6 million inhabitants. Isolates were collected from the territory where lives approximately more than 90% of the country’s population between January 2010 and December 2018. Invasive *S. pneumoniae* was defined as isolates obtained from blood, cerebrospinal fluid (CSF), pleural, peritoneal, joint fluid, and other normally sterile sites. The isolation and initial identification of *S. pneumoniae* were performed in the regional microbiological laboratories using conventional bacteriological techniques, such as colony appearance on blood agar, Gram staining, optochin susceptibility, and bile solubility, and the VITEK®2 system (bioMérieux, Marcy-l’Étoile, France) (Spellerberg et al., 2011).

All *S. pneumoniae* isolates, along with the clinical and demographic data (age, gender, date of admission to hospital, diagnosis, risk factors), were sent to the NRL for Streptococci at the Institute of Microbiology and Immunology, Faculty of Medicine, University of Belgrade. Patients were divided into five age groups: ≤ 2 years; >2– ≤ 5 years; >5–≤18 years; >18– < 65 years, and ≥ 65 years. After confirmation of identification by PCR detection of the *lytA* gene (Gillespie et al., 1994), isolates were stored in the STGG (skim milk, tryptone, glucose, glycerol) medium at −70°C until further analysis.

### 2.2. Serotyping and antimicrobial susceptibility testing

All *S. pneumoniae* isolates were serotyped by Quellung reaction, using pool, type, and factor-specific antisera (Statens Serum Institute, Copenhagen, Denmark).

Antimicrobial susceptibility of *S. pneumoniae* isolates to oxacillin, erythromycin, clindamycin, norfloxacin, tetracycline, chloramphenicol, trimethoprim-sulfamethoxazole, and vancomycin was determined by disk diffusion assay (Bio-Rad, United Kingdom), following the European Committee on Antimicrobial Susceptibility Testing (EUCAST) guidelines (Matuschek et al., 2014). The double disk diffusion method with erythromycin (15 μg) and clindamycin (2 μg) disks was used to detect phenotypes of macrolide resistance: the constitutive macrolide resistance (cMLS_B_)_,_ inducible macrolide resistance (iMLS_B_), and M phenotype. The minimum inhibitory concentrations (MICs) of penicillin, erythromycin, and clindamycin were determined using the MIC Test Strip (Liofilchem, Italy). *Streptococcus pneumoniae* ATCC 49619 was used as the control strain. Interpretation of susceptibility categories (S - susceptible, standard dosing regimen, I – susceptible, increased exposure, and R – resistant) was done using EUCAST guidelines (The European Committee on Antimicrobial Susceptibility Testing, 2022).

According to Mohanty et al. (2022), penicillin and ceftriaxone non-wild-type isolates, ie. *S. pneumoniae* with MIC to benzylpenicillin and ceftriaxone above those of wild-type isolates (>0.06 mg/L and > 0.5 mg/mL, respectively) should be analyzed and presented together, since they all have some level of resistance. All strains categorized as I and R for macrolides, fluoroquinolones, and trimethoprim-sulfamethoxazole were classified as non-susceptible (NS) (Lee et al., 2020).

Multidrug-resistant (MDR) isolates are defined as non-susceptible/resistant to three or more antimicrobial classes (Cillóniz et al., 2018). Extensive drug resistance (XDR) was defined as non-susceptibility/resistance to five or more of the following seven classes of antibiotics: β-lactams, macrolides, lincosamides, fluoroquinolones, chloramphenicol, tetracyclines, and folate-pathway inhibitors (Cillóniz et al., 2018).

### 2.3. Genetic determinants of macrolide resistance

All macrolide-resistant isolates were screened for macrolide-resistance genes. Total DNA from an overnight culture was extracted using a QIAamp DNA Mini Kit (QIAGEN GmbH, Hilden, Germany) according to the manufacturer’s instructions. All isolates were screened for *ermB* and *mefA* genes by PCR detection, as previously reported (El Ashkar et al., 2017).

### 2.4. Multilocus sequence typing

A total of 158 randomly selected *S. pneumoniae* isolates from all participating hospitals, with respect to the serotype frequency, relevance, and penicillin and macrolide resistance, were subjected to multilocus sequence typing (MLST). The MLST of the seven housekeeping genes (*aroE*, *gdh*, *gki*, *recP*, *spi*, *xpt*, and *ddl*) was conducted using previously described protocols (Enright and Spratt, 1998). STs were assigned according to the allelic profiles of 7 housekeeping genes using the MLST database^4^ (Jolley et al., 2018). In addition, a minimum spanning tree representing relationships between STs was generated using the geoBURST algorithm and visualized using PHYLOVIZ software^5^ (Feil et al., 2004). STs that shared at least five out of seven allelic variants were included in the same clonal complex (CC) (Setchanova et al., 2018). The clonal complexes were then assigned a unique identifier, represented by the ST of the most likely primary founder (central ST) within a clonal complex. Afterward, the STs obtained were compared with Pneumococcal Molecular Epidemiology Network (PMEN) clones^6^ to identify international antibiotic-resistant clones.

### 2.5. Statistical analysis

Statistical analysis was performed using SPSS version 20.0 (SPSS Inc., Chicago, IL, United States). For comparing categorical variables, Pearson’s chi-squared test or Fisher exact test was used. *p*-values of <0.05 were considered to be statistically significant.

## 3. Results

### 3.1. IPD notification rate, patients characteristics

During the nine-year period, a total of 490 invasive non-duplicate *S. pneumoniae* isolates were collected: 38 in 2010; 31 in 2011; 38 in 2012; 61 in 2013; 63 in 2014; 49 in 2015; 75 in 2016; 69 in 2017 and 64 in 2018. In the last 3 years of the study, during which the number of isolates per year stabilized, the notification rates were as follows: 1.14 (2016), 1.05 (2017), and 0.97 (2018) cases per 100,000 population. The age range of participants was 4 months to 92 years, with a median age of 53 (5-66) years. The representation of patients by age was as follows: ≤ 2 years, 101 (20.6%); >2– ≤ 5 years, 26 (5.3%); >5–≤18 years, 24 (4.9%); >18– < 65 years, 205 (41.8%); and ≥ 65 years, 134 (27.4%). Isolates were obtained from 288 males (58.8%) and 202 females (41.2%). *S. pneumoniae* isolates were recovered from blood culture (289; 59%), CSF (168; 34.3%), pleural fluid (30; 6.1%), and peritoneal fluid, pericardial fluid and joint fluid (one; 0.2% for each). In children ≤2 years, 74 (73.7%) strains were isolated from blood, and 26 (25.7%) from CSF, while in children >2 –≤18 years, 24 (48%), 20 (40%) and 6 (12%) were found in blood, CSF and pleural fluid, respectively. In adults (>18 years), 191 (56.3%), 122 (36%) and 24 (7.1%) strains were isolated from blood, CSF and pleural fluid, respectively. Occult bacteremia and septicemia were reported in 212 (43.3%), meningitis in 168 (34.3%), bacteremic pneumonia in 77 (15.7%), empyema pleurae in 30 (6.1%), and peritonitis, pericarditis, and arthritis in overall 3 (0.6%) cases.

### 3.2. Serotype distribution and coverage of PCV10, PCV13, and PPV23

Overall, 36 different serotypes were detected (26 in children and 35 in adults) in the present study, as depicted in Table 1. The most common pneumococcal serotypes among all isolates were 3 (96; 19.6%), 19F (59; 12.1%), 14 (53; 10.8%), 6B (35; 7.1%), followed by 6A (28; 5.7%), 19A (21; 4.3%) and 23F (21; 4.3%). Thirteen pneumococcal isolates (2.7%) were non-typable. Serotype 3 was significantly more frequent among adults compared to children (23.9% vs. 3%, *p* < 0.001), while serotypes 19F and 14 were significantly more common among children ≤2 years than among patients older than 2 years (18.8% vs. 10.3 and 17.8% vs. 9%, respectively, *p* < 0.05).

**Table 1:** Distribution of serotypes by age group among invasive *Streptococcus pneumoniae* isolates during 2010–2018.

Serotype (*N*)	Age categories, *N* (%)					≤2 years	>2– ≤ 5 years	>5– ≤ 18 years	>18– ≤ 65 years	>65 years
PCV10					
1 (5)	3 (3.0%)	0 (0%)	0 (0%)	1 (0.5%)	1 (0.7%)
4 (17)	1 (1.0%)	0 (0%)	1 (4.3%)	10 (4.9%)	5 (3.7%)
5 (1)	0 (0%)	1 (3.8%)	0 (0%)	0 (0%)	0 (0%)
6B (35)	16 (15.8%)	1 (3.8%)	1 (4.3%)	8 (3.9%)	9 (6.7%)
7F (17)	4 (4.0%)	1 (3.8%)	2 (8.7%)	8 (3.9%)	2 (1.5%)
9 V (10)	1 (1.0%)	0 (0%)	0 (0%)	5 (2.4%)	4 (3.0%)
14 (53)	18 (17.8%)	6 (23.1%)	2 (8.7%)	20 (9.8%)	7 (5.2%)
18C (15)	5 (5.0%)	2 (7.7%)	3 (13.0%)	4 (2.0%)	1 (0.7%)
19F (59)	19 (18.8%)	4 (15.4%)	3 (13.0%)	15 (7.3%)	18 (13.4%)
23F (21)	5 (5.0%)	0 (0%)	1 (4.3%)	5 (2.4%)	10 (7.5%)
PCV13					
3 (96)	3 (3.0%)	3 (11.5%)	2 (8.7%)	57 (27.8%)	31 (23.1%)
6A (28)	9 (8.9%)	3 (11.5%)	1 (4.3%)	6 (2.9%)	9 (6.7%)
19A (21)	3 (3.0%)	0 (0%)	4 (17.4%)	11 (5.4%)	3 (2.2%)
non-PCV13					
9 N (12)	1 (1.0%)	0 (0%)	1 (4.3%)	8 (3.9%)	2 (1.5%)
8 (11)	0 (0%)	0 (0%)	0 (0%)	7 (3.4%)	4 (3.0%)
11A (8)	1 (1.0%)	0 (0%)	0 (0%)	4 (2.0%)	3 (2.2%)
23A (8)	1 (1.0%)	2 (7.7%)	0 (0%)	3 (1.5%)	2 (1.5%)
10A (7)	1 (1.0%)	0 (0%)	1 (4.3%)	3 (1.5%)	2 (1.5%)
22F (7)	1 (1.0%)	0 (0%)	0 (0%)	3 (1.5%)	3 (2.2%)
9A (5)	1 (1.0%)	0 (0%)	0 (0%)	2 (1.0%)	2 (1.5%)
12F (5)	0 (0%)	1 (3.8%)	0 (0%)	1 (0.5%)	3 (2.2%)
15A (5)	0 (0%)	0 (0%)	0 (0%)	3 (1.5%)	2 (1.5%)
15C (5)	1 (1.0%)	0 (0%)	0 (0%)	3 (1.5%)	1 (0.7%)
15B (4)	0 (0%)	1 (3.8%)	0 (0%)	1 (0.5%)	2 (1.5%)
17F (4)	0 (0%)	0 (0%)	0 (0%)	2 (1.0%)	2 (1.5%)
33F (4)	1 (1.0%)	1 (3.8%)	1 (4.3%)	1 (0.5%)	0 (0%)
15F (3)	1 (1.0%)	0 (0%)	0 (0%)	2 (1.0%)	0 (0%)
20 (3)	1 (1.0%)	0 (0%)	0 (0%)	0 (0%)	2 (1.5%)
16F (2)	0 (0%)	0 (0%)	0 (0%)	1 (0.5%)	1 (0.7%)
6C (1)	0 (0%)	0 (0%)	0 (0%)	1 (0.5%)	0 (0%)
17A (1)	0 (0%)	0 (0%)	0 (0%)	1 (0.5%)	0 (0%)
18F (1)	0 (0%)	0 (0%)	0 (0%)	1 (0.5%)	0 (0%)
23B (1)	0 (0%)	0 (0%)	0 (0%)	0 (0%)	1 (0.7%)
31 (1)	0 (0%)	0 (0%)	0 (0%)	1 (0.5%)	0 (0%)
33A (1)	0 (0%)	0 (0%)	0 (0%)	1 (0.5%)	0 (0%)
NT (13)	4 (4.0%)	0 (0%)	1 (4.3%)	6 (2.9%)	2 (1.5%)
Total (490)	101 (100%)	26 (100%)	24 (100%)	205 (100%)	134 (100%)

The serotype distribution according to the patient’s age is shown in Table 1. Among the children younger than 2 years, the most common serotypes were 19F (18.8%), 14 (17.8%), 6B (15.8%), and 6A (8.9%). In the group >2 to ≤5 years, serotypes 19F (15.4%), 14 (23.1%), 6A, and 3 (11.5%, each) predominated. Serotypes 19A (17.4%), 19F, and 18C (13% each) were prevalent in children older than 5 years.

Among adults, the serotype distribution was similar in both age groups, with type 3 as the most common, 27.8% in >18- < 65 and 23.1% in ≥65. In adults less than 65, serotypes 14, 19F, and 19A were among the top five, accounting for 22.5% of all cases, while in older than 65, types 19F, 14, and 6A/6B were leaders with a total of 25.3% presentation.

**Figure 1:**
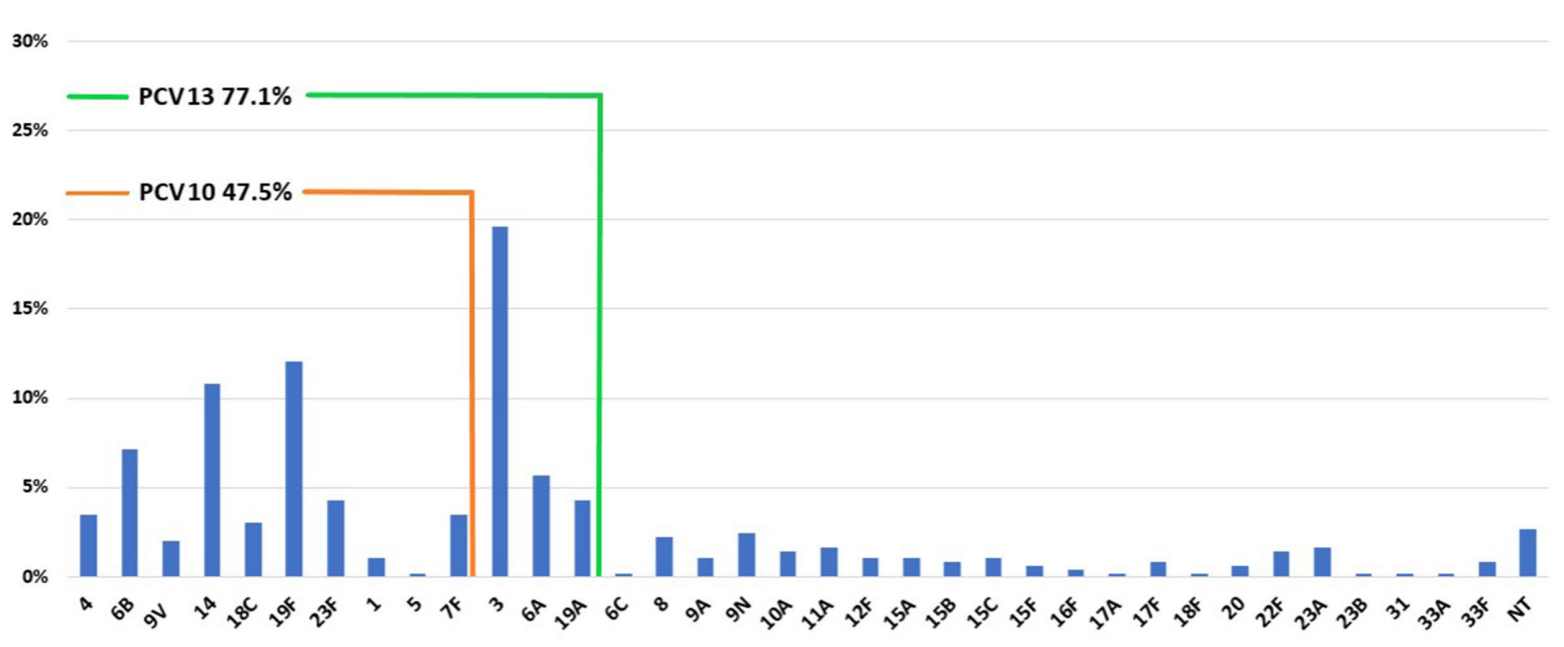
Distribution of serotypes and PCV10 and PCV13 coverage among pneumococci recovered from IPD during 2010–2018 in Serbia.

The coverage rate of PCV13 is considerably higher than PCV10 (Figure 1). In children under 2 years of age, PCV13 coverage was 86.1%, while PCV10 was 71.3% (Supplementary Figure 1). In older children (>2 - ≤5), the difference between PCV13 and PCV10 was higher (80.8% vs. 57.7%). In adults, PPV23 coverage was higher than PCV13 (for example, 85.1% vs. 74.6% in ≥65 years).

### 3.3. The trend of serotype distribution and vaccine coverage over the study period

The prevalence of seven common pneumococcal serotypes (represented by at least 20 isolates) for each year is shown in Supplementary Figure 2. During the study period, serotype 3 was the most prevalent type, with the exception of 2015, when 19F dominated. While the prevalence of serotype 3 increased from 18.5% in 2010 to 23.4% in 2018, 19F gradually decreased from 18.5 to 0% in 2018. A similar declining trend was observed for serotypes 14 and 23F (13 and 3.7% in 2010 vs. 4.7 and 0%, respectively). On the other hand, the prevalence of serotypes 6A, 6B, and 19A increased (1.9% each in 2010 to 3, 12.5, and 3.1%, respectively). The coverage rate of PCV10 among children younger than 5 years decreased over the study period, from 84.6% in 2010 to 53.3% in 2018 (Supplementary Table 1). On the contrary, PCV13 remained above 70% in the same age group, slowly decreasing from 84.6% in 2010 to 73.3% in 2018. The coverage rate of PPV23 in those older than 2 years remained stable with little variations during the whole time and above 80%, while PCV13 coverage in older than 5 years dropped from 70.7% in 2010 to 55.1% in 2018.

The most common serotypes (3, 19F, and 14) were isolated from blood and CSF with similar frequency. However, serotype 3 was more often found in pleural fluid in pleural empyema cases and bacteremic pneumonia compared to other types (*p* < 0.05). Conversely, 19F was less frequent among pneumonia cases than other types (*p* < 0.05).

### 3.4. Antimicrobial susceptibility and macrolide-resistance genes

Out of the 490 invasive *S. pneumoniae* isolates, 233 (47.6%) were PNSP [I = 104 (21.2%); *R* = 129 (26.3%)], as shown in Table 2. Among 81 CNSP, I and R categories were found in 12 (2.4%) and 69 (14.1%) isolates, respectively. Non-susceptibility rate to erythromycin was high and found in 40.4% of isolates [*I* = 2 (0.4%); *R* = 196 (40%)]. The most common phenotype of macrolide resistance was cMLSB 136 (68.7%), followed by M phenotype 54 (27.3%), whereas only 7 (3.5%) isolates showed iMLSB phenotype. MIC_50_ and MIC_90_ for penicillin were 0.094 mg/L and 2 mg/L, while MIC_50_ and MIC_90_ for erythromycin were 0.5 mg/L and 256 mg/L, respectively. The highest non-susceptibility was detected for tetracycline (34.9%) and trimethoprim/sulfamethoxazole (32%), whereas the lowest was found for chloramphenicol and fluoroquinolones - 10.8 and 1.4%, respectively. Resistance to vancomycin was not detected.

**Table 2:** Antimicrobial resistance of the seven most common *Streptococcus pneumoniae* serotypes.

Serotype (*N*)	Antibacterial agent *N* of non-susceptible isolates (%)											*P*	ER	P + ER	*C*	CL	FQ	TET	SXT	CHL	MDR	XDR
3 (96)	9 (9.4%)	5 (5.2%)	1 (1.0%)	2 (2.1%)	4 (4.2%)	0 (0%)	7 (7.3%)	4 (4.2%)	1 (1.0%)	2 (2.1%)	1 (1.0%)
19F (59)	47 (79.7%)	51 (86.4%)	44 (74.6%)	28 (47.5%)	50 (84.7%)	0 (0%)	49 (83.1%)	37 (62.7%)	3 (5.1%)	22 (37.3%)	29 (49.2%)
14 (53)	49 (92.5%)	34 (64.2%)	31 (58.5%)	15 (28.3%)	31 (58.5%)	2 (3.8%)	20 (37.7%)	45 (84.9%)	11 (20.8%)	15 (28.3%)	19 (35.8%)
6B (35)	34 (97.1%)	25 (71.4%)	24 (68.6%)	13 (37.1%)	24 (68.6%)	1 (2.9%)	25 (71.4%)	22 (62.9%)	17 (48.6%)	9 (25.7%)	19 (54.3%)
6A (28)	23 (82.1%)	21 (75.0%)	18 (64.3%)	3 (10.7%)	5 (17.9%)	2 (7.1%)	7 (25.0%)	3 (10.7%)	0 (0%)	6 (21.4%)	1 (3.6%)
19A (21)	9 (42.9%)	8 (38.1%)	6 (28.6%)	4 (19.0%)	6 (28.6%)	0 (0%)	8 (38.1%)	7 (33.3%)	0 (0%)	7 (33.3%)	2 (9.5%)
23F (21)	17 (81.0%)	12 (57.1%)	11 (52.4%)	7 (3.3%)	4 (19.0%)	0 (0%)	15 (71.4%)	10 (47.6%)	8 (38.1%)	8 (38.1%)	7 (3.3%)
Other (177)	45 (25.4%)	42 (23.7%)	25 (14.1%)	9 (5.1%)	20 (11.3%)	2 (1.1%)	40 (22.6%)	29 (16.4%)	13 (7.3%)	28 (15.8%)	6 (3.4%)
Total (490)	233 (47.6%)	198 (40.4%)	160 (32.7%)	81 (16.5%)	144 (29.4%)	7 (1.4%)	171 (34.9%)	157 (32.0%)	53 (10.8%)	98 (20.0%)	83 (16.9%)

Non-susceptible, I (susceptible, increased exposure) and R (resistant) isolates; P, penicillin; ER, erythromycin; C, ceftriaxone; CL, clindamycin; FQ, fluoroquinolones; TET, tetracycline; SXT, trimethoprim-sulfamethoxazole; CHL, chloramphenicol; MDR, multidrug-resistant; XDR, extensive drug resistance.

Simultaneous non-susceptibility to erythromycin and penicillin was found in 32.7% of the isolates (Table 2). MDR was detected in 98 (20.0%) isolates belonging to 24 different serotypes (3, 8, 20, 11A, 12F, 14, 15A, 15B, 15C, 15F, 17F, 18C, 19A, 19F, 22F, 23A, 23F, 33F, 6A, 6B, 6C, 7F, 9A, and 9 V), while XDR was found in 83 (16.9%) isolates, belonging to 12 different serotypes (3, 11A, 14, 15A, 19A, 19F, 22F, 23A, 23F, 6A, 6B, and 9A). MDR and XDR isolates were more commonly isolated from children younger than 2 years compared to other age groups (54.5% vs. 32.4%, *p* < 0.05). Non-susceptibility to penicillin/ceftriaxone, erythromycin, tetracycline, and clindamycin was the most common resistance pattern among MDR pneumococci (27/98), while non-susceptibility to penicillin/ceftriaxone, erythromycin, tetracycline, clindamycin, and trimethoprim-sulfamethoxazole (41/83) was most frequent among XDR (Supplementary Table 2).

Overall, predominant serotype 3 was significantly (*p* < 0.01) the most susceptible with the lowest rate of PNSP (9.4%), erythromycin (5.2%), simultaneous non-susceptibility to penicillin and erythromycin (1%), MDR (2.1%) and XDR (1.0%). The highest prevalence of PNSP was found in serotypes 6B (97.1%), 14 (92.5%), and 6A (82.1%). Serotypes 19F, 6B, and 6A were highly co-resistant simultaneous non-susceptible to penicillin and erythromycin (74.6, 68.8, and 64.3%, respectively). The highest proportion of MDR was found in 23F (38.1%), 19F (37.3%), and 19A (33.3%), whereas XDR isolates were most common in 6B (54.3%), 19F (49.2%) and 14 (35.8%) serotypes. Overall, the most resistant serotypes were 19F, 14, and 6B, which were significantly more resistant to tested antibiotics with more MDR and XDR representatives (p < 0.01).

Among the isolates with cMLSB phenotype, 93 (68.4%) isolates harbored only the *ermB*, two (1.5%) isolates harbored only *mefA* gene, while 33 (24.3%) isolates harbored both the *ermB* and *mefA* genes. Among isolates with iMLSB phenotype, the *ermB* gene was detected in one isolate, while the *mefA* gene was detected in four isolates. No genes were detected in 10 isolates – two isolates with the iMLSB phenotype and 8 (5.8%) isolates with the cMLSB phenotype. The *mefA* gene was detected in 52 (96.3%) isolates with *M* phenotype.

### 3.5. Multilocus sequence typing

**Figure 2:**
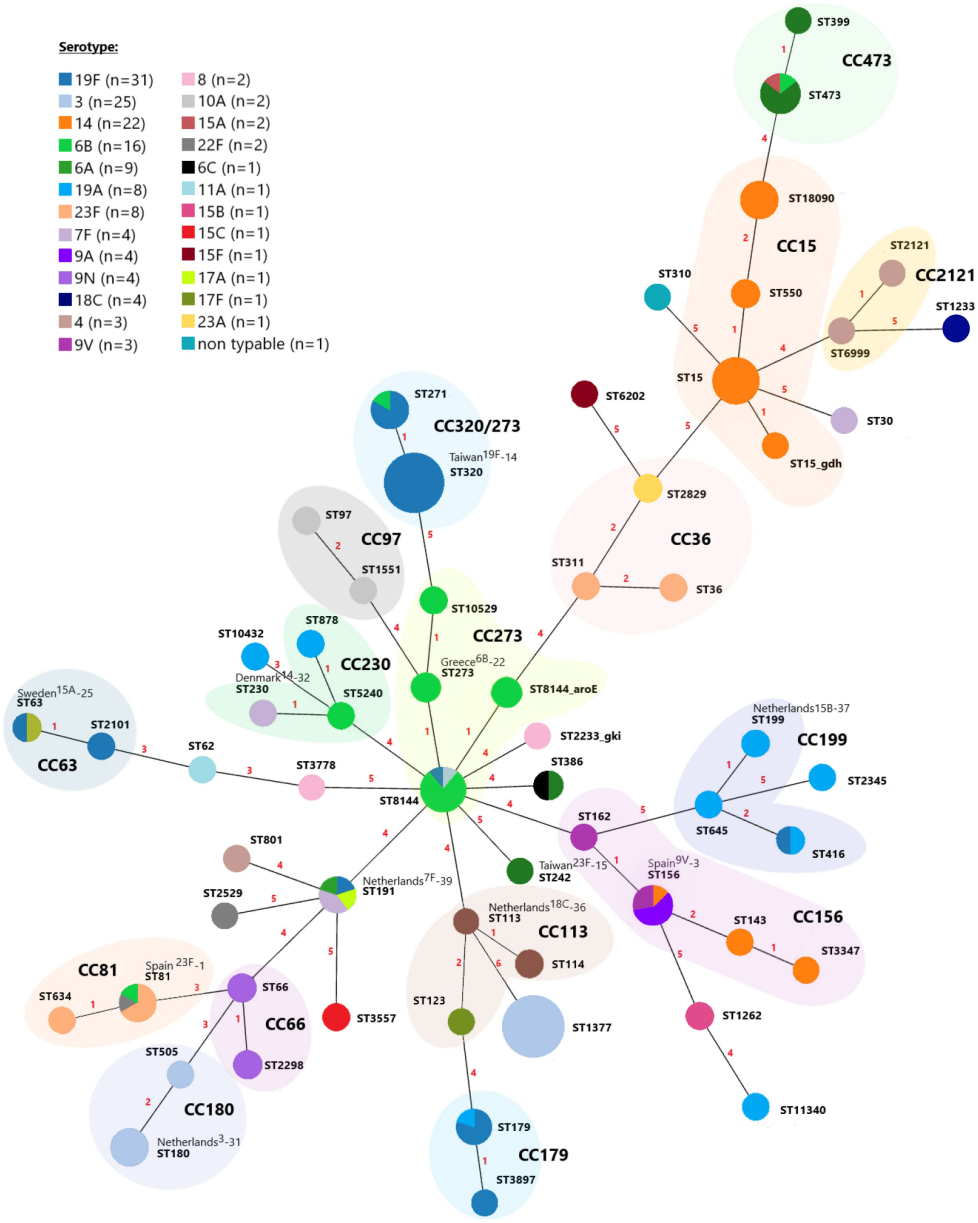
A minimum spanning tree of MLST for 158 invasive *Streptococcus pneumoniae* isolates over the nine-year period. Each circle represents a Sequence Type (ST), and the circle’s color represents the detected serotype. The area of each circle corresponds to the number of isolates. The numbering shown between the connected nodes indicates the differences in loci between the MLST profiles, and pastel zones between some groups of circles indicate that these profiles belong to the same clonal complex (CC).

The Multilocus sequence typing (MLST) analysis of 158 pneumococci identified 60 different STs belonging to the 16 Clonal Complexes (CCs) (consisting of 42 STs) and 18 singletons (Figure 2). Among the 60 STs identified, 3 (5%) were new in the PubMLST website database, one was presented in our population as a singleton, and two were grouped in CCs (Figure 2). Seven CCs and one STs (ST1377, CC320, CC15, CC273, CC156, CC473, CC81, and CC180) represented 65.8% (*n* = 104) of all isolates. Several STs were associated with more than one serotype (Figure 2): ST63 (19F and 15A), ST81 (23F, 22F, and 6B), ST156 (14, 9 V, and 9A), ST179 (19A and 19F), ST191 (6A, 7F, 17A, and 19F), ST271 (19F and 6B), ST386 (6A and 6C), ST416 (19A and 19F), ST473 (6A, 6B, and 15A) and ST8144 (3, 6B, and 19F).

Most serotypes were associated with more than one CC (Supplementary Table S3). The majority of serotype 19F isolates belong to the CC320/271 (21/31, 67.7%), followed by CC179 (5/31, 16.1%). The ST1377 represented 68% (17/25) of the serotype 3 isolates, followed by CC180 (7/25, 28%) and CC273 (1/25, 4%). The main clonal complexes among serotype 14 isolates were CC15 (19/22, 86.4%) and CC156 (3/22, 13.6%). Of the clonal complexes identified among serotype 6B isolates, CC273 was the largest and represented 80% (12/16) of the isolates. For serotype 6A, CC473 was identified as most prevalent (6/9, 66.6%), while three isolates belonged to the singleton STs (ST191, ST242, and ST386). Among the serotype 19A isolates, three common clonal complexes were identified, CC199 (3/8, 37.5%), CC179 (1/8, 12.5%), and CC230 (1/8, 12.5%). Three isolates belonged to the singleton STs (2,345, ST10432, and ST11340). Serotype 23F isolates differentiated into two clonal complexes, CC242 (5/8, 62.5%) and CC156 (3/8, 37.5%).

A total of 11 international clones or their single locus variants (SLVs) or double locus variants were discovered among 16 different STs, including Spain23F-1, Spain9V-3, Taiwan19F-14, Taiwan23F-15, Greece6B-22, Sweden15A-25, Netherlands3-31, Denmark14-32, Netherlands18C-36, Netherlands15B-37 and Netherlands7F-39 (Supplementary Table S3).

### 3.6. Relationship between antimicrobial susceptibility and clonal complexes

Table 3 summarizes the analysis of antibiotic resistance profiles according to CC/STs. All isolates belonging to CC15, CC81, CC179, CC230, and CC273 were PNSP, with high rates of CNSP (CC15, 42.1%; CC81, 57.1%; CC179, 100%; CC230, 66.6%; CC273, 50%). High rates of non-susceptibility resistance to erythromycin, clindamycin, tetracycline, and trimethoprim/sulfamethoxazole (up to 100%) were also detected among these isolates (Table 3).

**Table 3:** Non-susceptibility rate of the *Streptococcus pneumoniae* isolates grouped by clonal complex/sequence type to penicillin, ceftriaxone, erythromycin, clindamycin, tetracycline, trimethoprim/sulfamethoxazole, and chloramphenicol.

Clonal complex	Antibacterial agent *N* of non-susceptible isolates (%)								P	C	ER	CL	TET	SXT	CHL	MDR
ST1377 (*n* = 17)	1 (5.9%)	0 (0%)	0 (0%)	0 (0%)	1 (5.9%)	0 (0%)	0 (0%)	0 (0%)
CC15 (*n* = 19)	19 (100%)	8 (42.1%)	14 (73.7%)	14 (73.7%)	8 (42.1%)	19 (100%)	5 (26.3%)	14 (73.7%)
CC36 (*n* = 4)	2 (50%)	1 (25%)	1 (25%)	0 (0%)	2 (50%)	1 (25%)	0 (0%)	1 (25%)
CC63 (*n* = 3)	2 (66.6%)	0 (0%)	3 (100%)	3 (100%)	3 (100%)	0 (0%)	0 (0%)	3 (100%)
CC66 (*n* = 4)	1 (25%)	0 (0%)	0 (0%)	0 (0%)	2 (50%)	0 (0%)	0 (0%)	0 (0%)
CC81 (*n* = 7)	7 (100%)	4 (57.1%)	5 (71.4%)	0 (0%)	7 (100%)	6 (85.7%)	6 (85.7%)	7 (100%)
CC97 (*n* = 2)	0 (0%)	0 (0%)	0 (0%)	0 (0%)	0 (0%)	0 (0%)	0 (0%)	0 (0%)
CC113 (*n* = 4)	1 (25%)	0 (0%)	3 (75%)	0 (0%)	0 (0%)	1 (25%)	0 (0%)	0 (0%)
CC156 (*n* = 10)	9 (90%)	2 (20%)	9 (90%)	3 (30%)	9 (90%)	6 (60%)	1 (10%)	9 (90%)
CC179 (*n* = 6)	6 (100%)	6 (100%)	6 (100%)	6 (100%)	6 (100%)	1 (16.7%)	0 (0%)	6 (100%)
CC180 (*n* = 7)	0 (0%)	0 (0%)	1 (14.3%)	1 (14.3%)	1 (14.3%)	0 (0%)	0 (0%)	1 (14.3%)
CC199 (*n* = 4)	1 (25%)	0 (0%)	3 (75%)	3 (75%)	3 (75%)	1 (25%)	0 (0%)	3 (75%)
CC230 (*n* = 3)	3 (100%)	2 (66.7%)	3 (100%)	2 (66.7%)	3 (100%)	2 (66.6%)	1 (33.3%)	3 (100%)
CC273 (*n* = 14)	14 (100%)	7 (50%)	12 (85.7%)	12 (85.7%)	12 (85.7%)	12 (85.7%)	10 (71.4%)	13 (92.9%)
CC473 (*n* = 8)	7 (87.5%)	0 (0%)	7 (87.5%)	0 (0%)	0 (0%)	0 (0%)	0 (0%)	0 (0%)
CC2121 (*n* = 2)	0 (0%)	1 (50%)	0 (0%)	0 (0%)	1 (50%)	0 (0%)	0 (0%)	0 (0%)
CC320/271 (*n* = 22)	21 (95.5%)	14 (63.6%)	22 (100%)	22 (100%)	20 (91%)	22 (100%)	0 (0%)	22 (100%)
Others (*n* = 22)	9 (40.9%)	1 (4.5%)	8 (36.4%)	4 (18.2%)	9 (40.9%)	4 (18.2%)	0 (0%)	8 (36.4%)

Non-susceptible, I (susceptible, increased exposure) and R (resistant) isolates; P, penicillin; ER, erythromycin; C, ceftriaxone; CL, clindamycin; FQ, fluoroquinolones; TET, tetracycline; SXT, trimethoprim-sulfamethoxazole; CHL, chloramphenicol; MDR, multidrug-resistant.

The PNSP rates of the majority of isolates belonging to CC63, CC156, CC473, and CC320/271 ranged from 66.6 to 95.5%. In addition, only genotype ST320/271 expressed a CNSP rate of 63.6%. Moreover, all isolates of the CC63, CC81, CC179, CC230, and CC320/271, and majority of CC15, CC156, CC199 and CC273, were categorized as MDR.

## 4. Discussion

This is the first comprehensive nationwide study on the characterization of circulating clones of invasive *S. pneumoniae* isolates in Serbia. Data from this study provide a baseline for future studies monitoring the impact of PCVs on serotype distribution and provide recommendations for necessary changes in the immunization schedule. They also shed light on the coverage of PPV23, a vaccine mandatory for certain patient categories since 2020.^7^

According to the NRL-based surveillance, the annual notification rate of IPD varies between 0.97 and 1.14 cases per 100,000 population in the last three years of the study. Observed rate is much lower than in the Netherlands (16), Sweden (13.9), Slovenia (13.4), and Norway (11.0) but higher than in Bulgaria (0.3), Romania, and Greece (each 0.4) and Croatia (0.5) in 2018 (ECDC, 2018). There are several reasons why IPD incidence is certainly underestimated in Serbia: primarily due to the voluntary-based reporting of IPD, insufficient blood sampling for blood culture, starting antibiotic therapy before sample collection, suboptimal standard culture techniques, voluntary sending of bacterial isolates, etc. Consequently, NRL receives a relatively small number of invasive pneumococcal isolates per year. Therefore, there is a clear need for improvement of blood specimen collection practices and IPD surveillance in our country because, despite the legislative obligation, it is poorly implemented. Also, the introduction of molecular methods in the routine practice would certainly improve diagnosis and surveillance of IPD. Since the detection of *the lytA* gene is not absolutely specific to *S. pneumoniae* (Tavares et al., 2019), the combination of target genes (*psaA, wzg/cpsA*, etc.) has been suggested to improve the reliability of the identification.

IPD was predominantly reported in adults (69.2%), particularly in persons between 18 and 65 years old (41.8%) and children ≤2 years old (20.6%). Obtained results are not in line with the report of the European Centre for Disease Prevention and Control (ECDC) for 2018 (ECDC, 2018), where the highest incidence of IPD was among patients ≥65 years. However, it is in accordance with the findings of Marrie T et al., who reported that 27.3 and 53.5% of the IPD cases were in persons aged ≥65 and 17-54 years, respectively (Marrie et al., 2018). On the other hand, the percentage of isolates in infants (20.6%) and the elderly ≥65 years (27.4%) correlates well with ECDC data (ECDC, 2018). Blood was the most common specimen source (59%) obtained from cases of septicemia/occult bacteriemia and bacteremic pneumonia, followed by CSF (34.3%) taken from meningitis cases and pleural fluid (6.1%) collected from patients with pleural empyema. ECDC reported slightly different results, indicating that the most common clinical presentations of IPD in European countries were septicemia (35%), bacteremic pneumonia (43%), and meningitis (19%) (ECDC, 2018). The discrepancy in the incidences of meningitis cases between our and EU data may be due to the fact that clinical presentation was known for 34% of all IPD cases in the EU and that PCVs have been introduced in EU countries before than Serbia, resulting in the lower incidence of IPD in infants and decline in meningitis cases.

In the present study, almost two-thirds of all pneumococcal isolates belonged to the most common seven serotypes (3, 19F, 14, 6B, 6A, 19A, and 23F), which corresponds to the pre-PCV period in most European countries, such as Finland (14, 3, and 23F), Bulgaria (3 and 19F), Slovenia (14, 9 V, 3, 7F, 19A, and 1) and the United States (6B, 14, 19F, 23F, 3, 6A, and 19A) (Setchanova et al., 2012; Richter et al., 2013; Jokinen et al., 2015; Müller Premru et al., 2015).

Over the nine-year study period, the coverage rate of PCV10 among children <5 years declined from 84.6% in 2010 to 53.3% in 2018, while the coverage of PCV13 slightly decreased from 84.6% in 2010 to 73.3% in 2018. Also, the coverage rate of PPV23 in those older than 2 years was quite stable (84.1% in 2010 vs. 80.8% in 2018). Immediately after the introduction of vaccination, in the second decade of the 2000s, the coverage rate of PCV10 and PCV13 on all IPD cases in Austria, Netherlands, and Bulgaria were 71 and 93%, 42, and 60%, 59.9 and 78.8% (Setchanova et al., 2012; Netherlands Reference Laboratory for Bacterial Meningitis (AMC/RIVM), 2014; Paulke-Korinek et al., 2014).

Unlike in most countries, a high incidence of serotype 3 was registered in the pre-vaccine period in Serbia. Reports from countries that have used PCV10 indicated a remarkable increase in PCV13 non-PCV10 serotypes, particularly 3 and 19A. Although some studies (Domingues et al., 2014; Deceuninck et al., 2015; Jokinen et al., 2015) suggest that PCV10 may provide cross-protection against 19A IPD in young children, no reduction in serotype 19A-related IPD was seen in older children and in adults. In fact, serotype 19A became significantly and increasingly prevalent, particularly in PCV10 countries. Thus, in Austria, serotype 19A increased from 3% of all IPD cases in the pre-PCV to 6–7% in just 2 years (Paulke-Korinek et al., 2014). Also, in Finland, 4 years after PCV10 introduction, the most prevalent serotypes were 19A (30%) and 3 (19%) (Jokinen et al., 2015). Further studies should evaluate the potential increase in these serotypes in Serbia.

It should be noted that IPD coverage of PPV23 in our adult population is high and exceed 85%, as it was provided that this vaccine could provide moderate long-term protection against hospitalization with PPV23 serotype pneumonia and IPD (Lawrence et al., 2020).

As the consumption of antibiotics in Serbia is still very high, especially broad-spectrum macrolides, quinolones, and third-generation cephalosporins (Tomas et al., 2021), bacterial resistance is on the rise. Results on antimicrobial resistance of invasive *S. pneumoniae* are in line with previous findings that reported high levels of pneumococcal non-susceptibility to penicillin (47.6%) and macrolides (40.4%) in Serbia (Gajic et al., 2013; Delic et al., 2021). Particularly worrying is the non-susceptibility to the third-generation cephalosporins (16.6%) and high levels of MDR (20%) and XDR (16.9%) isolates, which mainly express simultaneous non-susceptibility to first-line anti-pneumococcal antibiotics. Observed macrolide non-susceptibility was 40.4%, mainly due to the cMLSB-phenotype (68.7%), which is a similar rate as in a number of European countries, including Belgium, France, and Spain in the pre-vaccine era, with an average rate of 62.3% (Farrell et al., 2008). In contrast, the M-phenotype associated with the *mef* genes was the predominant resistance mechanism in some European countries (Austria, Bulgaria, Ireland, Finland, Germany, Greece, United Kingdom) and the United States in the pre-PCV period (Farrell et al., 2008; Siira et al., 2009; Bley et al., 2011; Setchanova et al., 2012). In the present study, the simultaneous presence of both *mef* and *erm* genes was detected in 16.6% of the isolates, which is higher than the mean European value (6.1%) at that time (Berbel et al., 2022). However, 5% of the tested macrolide-resistant isolates were negative for both genes, indicating the possibility of other resistance mechanisms such as L4 and L22 ribosomal protein mutations, or the presence of other *mef* and *erm* gene variants (Nagai et al., 2002; Midouni Ayadi et al., 2020), but additional research is warranted. The rates of pneumococcal non-susceptibility to penicillin and macrolides in Serbia were generally higher than in most European countries in the period 2016-2018, such as Austria, Czech Republic, Netherlands, Germany, United Kingdom, France, Spain, Poland, and Italy (WHO Regional Office for Europe/European Centre for Disease Prevention and Control, 2022). However, it is comparable with those found in Belarus, Bosnia and Herzegovina, Cyprus, France, Iceland, Malta, Romania, and Turkey, where the non-susceptibility rates to penicillin and macrolides were above 25% (WHO Regional Office for Europe/European Centre for Disease Prevention and Control, 2022).

In the current study, the most resistant serotypes were 14, 19F, 6B, 6A, and 23F, which is comparable with results reported in other European countries and the USA before PCVs introduction (Dagan and Klugman, 2008). Serotype 19A has been typically associated with antibiotic resistance (Desmet et al., 2022); in this study, we did not find significant resistance in our 19A isolates. However, due to the high coverage of PCV10 and PCV13 of resistant *S. pneumoniae* isolates (74.6 and 85.1%, respectively), a decline in pneumococcal resistance in our country could be expected.

This study revealed circulating CCs of invasive pneumococcus in Serbia for the first time. The majority of the detected STs in Serbia have also been reported in the pre-PCV period worldwide (ST62, ST81, ST180, ST199, ST271, ST320) and in neighboring countries (ST15, ST66, ST156, ST191, ST230, ST473, ST1377) (Müller Premru et al., 2015; Li et al., 2019; Sheppard et al., 2019).^8^ In the present study, 44.3% of the detected clones were closely related to 11 of the 43 PMEN clones (Spain23F-1, Spain9V-3, Taiwan19F-14, Taiwan23F-15, Greece6B-22, Sweden15A-25, Netherlands3-31, Denmark14-32, Netherlands18C-36, Netherlands15B-37, and Netherlands7F-39). ST1377 was the most common genotype among serotype 3 isolates, while globally disseminated Netherlands3-31 (ST180) only represented 25% of our isolates.

As observed in some countries before the introduction or widespread use of PCV7, the MDR 19A serotype pneumococci were also identified in our population (Choi et al., 2008). The *S. pneumoniae* 19A isolates were genetically heterogenous and were assigned to eight different STs (ST179, ST199, ST416, ST645, ST878, ST2345, ST10432, and ST11340). However, the globally disseminated MDR CC320/271 genotype responsible for the post-conjugate vaccine increase in MDR serotype 19A (Ruiz García et al., 2021) represented 74.2% of our serotype 19F isolates, all of which were MDR. Similar findings were reported by Ndlangisa et al., where no 19A serotype isolates were detected among CC320/271 (Ndlangisa et al., 2014).

While exceptions exist, it is generally observed that isolates sharing the same ST or CC tend to exhibit the same serotype (Brueggemann et al., 2003). Therefore, a new or unusual serotype-sequence type combination could indicate capsular switching. Among our isolates, we identified new ST-serotype combinations. Based on the PubMLST database, ST230 is associated with multiple serotypes, but it has not been reported among serotype 7F isolates; however, one of our serotype 7F isolates was ST230. Similarly, among CC320/271 isolates commonly associated with serotypes 19A and 19F, one isolate with 6B serotype was detected.

This study has several limitations. Firstly, the isolates collected in this study were sent on a voluntary basis, and the number of isolates causing IPD is probably underestimated. Secondly, only randomly selected isolates were genotyped by MLST due to financial limitations.

## 5. Conclusion

Overall, our study provides a comprehensive insight into the epidemiological diversity of *S. pneumoniae* isolates causing IPD in Serbia before PCV introduction into NIP. Serotype 3 is dominant in adults, while 19F and 14 were the most common in children. The PCV10 and PCV13 may provide significant protection to our children ≤2 years, with 71.3 and 86.1% coverage, respectively. Our isolates are highly non-susceptibile to penicillin and macrolides, with worrisome rates of MDR and XDR strains. Substantial coverage of implemented PCVs should provide protection against invasive diseases and MDR strains. Also, there is considerable diversity among the isolates, with 60 circulating STs detected. Therefore, the distribution of serotypes, antimicrobial resistance, and clonal association of invasive pneumococcal strains as a response to PCV implementation should be further monitored.

## Data availability statement

The original contributions presented in the study are included in the article/Supplementary material, further inquiries can be directed to the corresponding author.

## Author contributions

NO and IG contributed to the conception and design of the study. DK, MJ, ZV, DM, TT, SL, and JK were involved in the laboratory work and investigation. MJ and JK organized the database, prepared the figures and tables, and conducted the statistical analysis. NO, MJ, and JK wrote the sections of the manuscript. LR coordinated the study. All authors have read and agreed to the published version of the manuscript.

## Funding

This work was supported partly by a research grant from Investigator-Initiated Studies Program of Merck, Sharp & Dohme doo (grant number 60165) and by the Ministry of Education, Science, and Technological Development of the Republic of Serbia, Serbia [grant No. 451-03-68/2022-14/200110]. The opinions expressed in this paper are those of the authors and do not necessarily represent those of Merck, Sharp & Dohme doo.

## Conflict of interest

NO acts as a principal investigator for investigator-initiated sponsored research related to the topic conducted on behalf of the Faculty of Medicine, University of Belgrade, for which the Faculty obtained a research grant from Merck Sharp and Dohme doo. There are no patents, products in development, or marketed products associated with this research. We fully adhere to Frontiers policies on data and material sharing.

The remaining authors declare that the research was conducted in the absence of any commercial or financial relationships that could be construed as a potential conflict of interest.

## Publisher’s note

All claims expressed in this article are solely those of the authors and do not necessarily represent those of their affiliated organizations, or those of the publisher, the editors and the reviewers. Any product that may be evaluated in this article, or claim that may be made by its manufacturer, is not guaranteed or endorsed by the publisher.

## Footnotes

^1^Available at: https://www.pravno-informacioni-sistem.rs/SlGlasnikPortal/eli/rep/sgrs/ministarstva/pravilnik/2017/112/1/reg (In Serbian).

^2^Available at: https://www.batut.org.rs/download/izvestaji/Godisnji%20imunizacija%202018.pdf.

^3^Available at: https://www.paragraf.rs/propisi/zakon_o_zastiti_stanovnistva_od_zaraznih_bolesti.html (In Serbian).

^4^ https://pubmlst.org/spneumoniae

^5^ http://www.phyloviz.net/goeburst/

^6^Available at: https://www.pneumogen.net/gps/pmen.html.

^7^Available at: https://www.paragraf.rs/propisi/pravilnik-o-programu-obavezne-i-preporucene-imunizacije-stanovnistva-protiv-odredjenih-zaraznih-bolesti.html.

^8^Available at: https://data.monocle.sanger.ac.uk/.
