# Supplementary material for: Serotype distribution, antimicrobial susceptibility and molecular epidemiology of invasive Streptococcus pneumoniae in the nine-year period in Serbia
Source: Front Microbiol. 2023 Aug 21;14:1244366. doi: 10.3389/fmicb.2023.1244366 (PMC10475725; doi:10.3389/fmicb.2023.1244366)
Supplement: Supplementary file 2 [file Data_Sheet_1.docx]

Supplementary Material

Serotype distribution, antimicrobial susceptibility and molecular epidemiology of invasive *Streptococcus pneumoniae* in the nine-year period in Serbia

Natasa Opavski*, Milos Jovicevic, Jovana Kabic, Dusan Kekic, Zorica Vasiljevic, Tanja Tosic, Deana Medic, Suzana Laban, Lazar Ranin, Ina Gajic

*** Correspondence:** Natasa Opavski, natasaopavski@gmail.com

**Supplementary Figures**


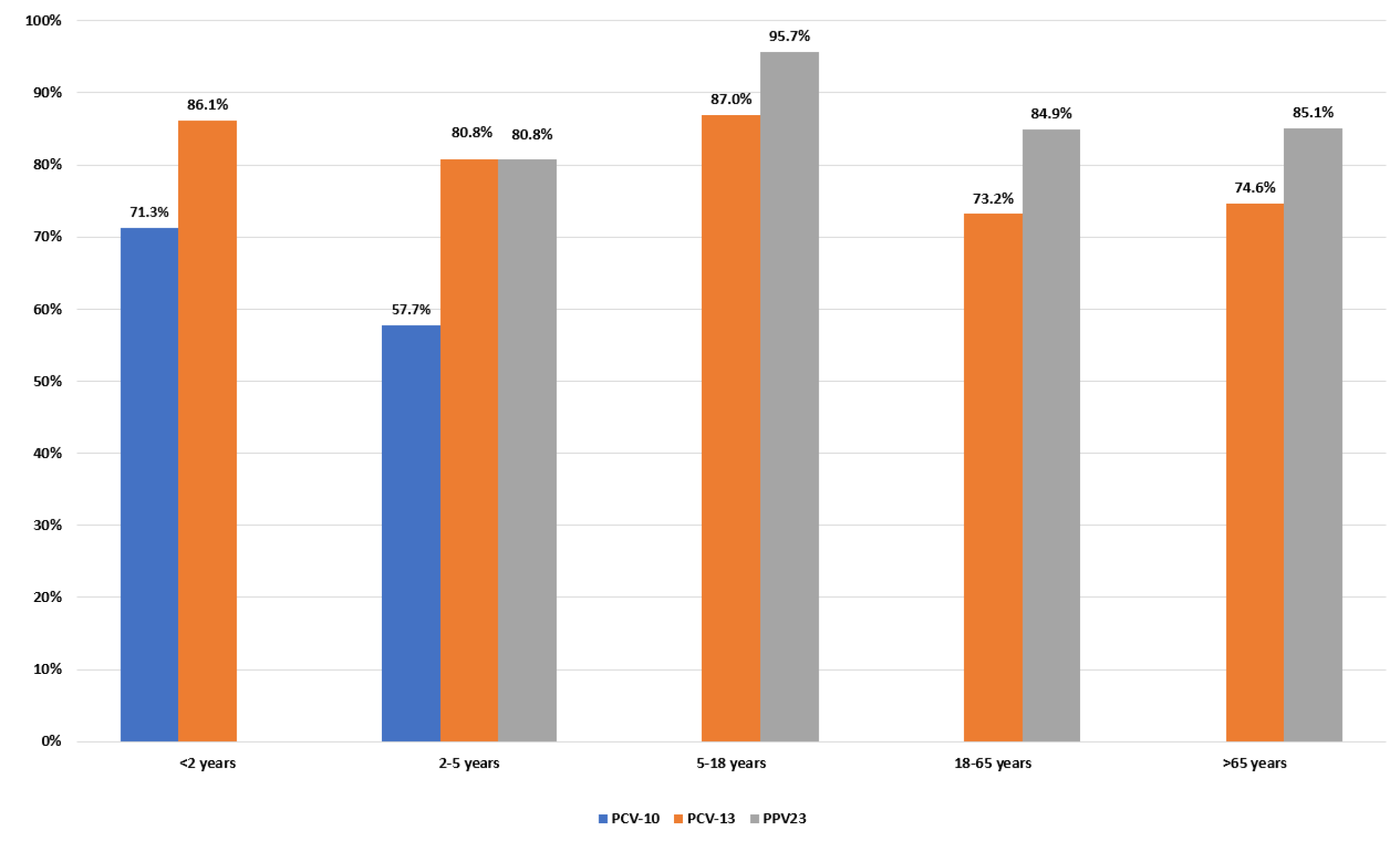
**Supplementary Figure 1:** PCV10, PCV13, and PPV23 coverage rates among different age groups.


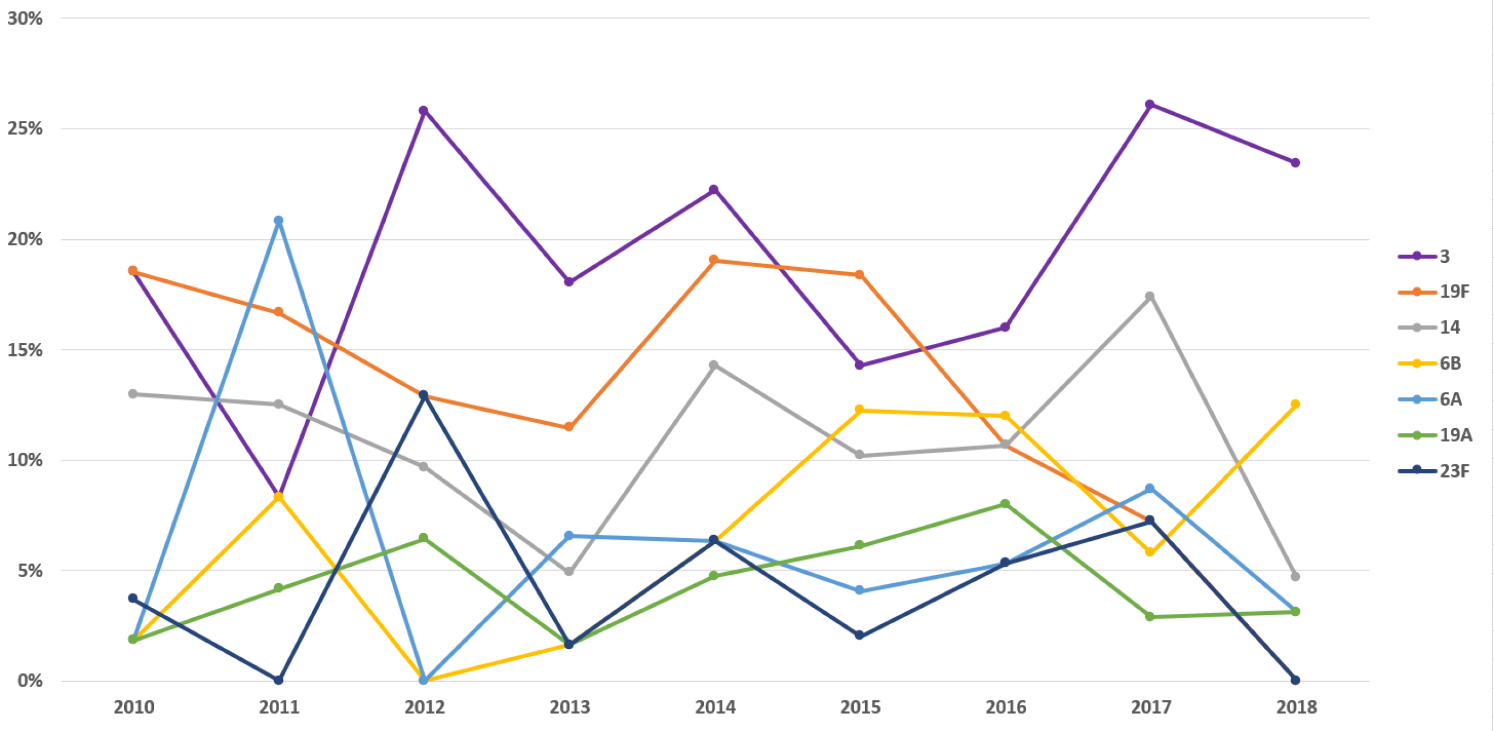
**Supplementary Figure 2**. Changes of frequencies of most common serotypes among *Streptococcus pneumoniae* strains during 2010– 2018 in Serbia.

**Supplementary Tables Captions**

**Supplementary Table 1.** Changes in overage rates of PCV10, PCV13, and PPV23 during the study period among target age groups.

**Supplementary Table 2**. Detected resistance patterns among invasive multidrug-resistant and extensive drug-resistant *Streptococcus pneumoniae* isolates.

**Supplementary Table 3**. Detected circulating clonal complexes among 158 invasive *Streptococcus pneumoniae* isolates from Serbia.
